# Severe Maternal Morbidity with the Inclusion of Events after Delivery Hospitalization

**DOI:** 10.1055/a-2817-3616

**Published:** 2026-03-18

**Authors:** Karen L. Schneider, Keri L. Calkins, Patrick S. Romano, Judy George, Whitney Schott, Leanna S. Sudhof

**Affiliations:** 1 Nutrition, Health, and Human Services Division, Mathematica, Inc., Princeton, New Jersey, United States; 2 Division of General Medicine and Bioethics, Department of Internal Medicine and Center for Healthcare Policy and Research, University of California, Davis, Sacramento, California, United States; 3 Quality Indicators Program, Agency for Healthcare Research and Quality, Rockville, Maryland, United States; 4 Department of Obstetrics and Gynecology, University of California, Davis, Sacramento, California, United States

**Keywords:** maternal health, severe maternal morbidity, obstetrics, quality measure, postpartum period, fourth trimester

## Abstract

**Objective:**

Severe maternal morbidity (SMM) is a growing public health concern in the United States. While existing measures capture SMM-related complications during the delivery hospitalization, patients may also experience serious complications after discharge that affect long-term health, mortality, and health care utilization. We aimed to assess the frequency of SMM events occurring after hospital discharge and identify the appropriate postdischarge window for measurement.

**Study Design:**

We analyzed 2019 to 2021 delivery hospitalizations among women aged 12 to 55 years using the Agency for Healthcare Research and Quality (AHRQ) Healthcare Cost and Utilization Project State Inpatient Databases and State Emergency Department Databases from 17 states, with follow-up through 2021. SMM was identified per AHRQ measure specifications in inpatient and emergency department encounters. We calculated overall SMM rates as well as specific SMM-related complications, during delivery hospitalization and after discharge.

**Results:**

The SMM rate during delivery hospitalization was 87.1 per 10,000 deliveries. Extending the measurement period through 42 days' postdischarge increased the cumulative SMM rate by 32.1% to 115.0 per 10,000 deliveries. More than 80% of SMM events observed within 90 days occurred in the first 42 days after discharge, and 78.6% of these were treated in inpatient settings. Coagulopathy (26.0 per 10,000), acute renal failure (21.4 per 10,000), and sepsis (25.3 per 10,000) had the highest cumulative rates through 42 days' postdischarge. Coagulopathy was the most common complication during the delivery hospitalization (27.7%), whereas sepsis emerged as the most frequent condition treated in the 42 days after discharge (34.4%).

**Conclusion:**

Our study highlights the importance of including the postpartum period when measuring SMM. Most events were treated in an inpatient setting, and the majority occurred within 42 days after delivery. To address SMM, research and policy warrants focus on maternal health during and after the delivery hospitalization.

**Key Points:**

- Including events through 42 days' postdischarge increased the cumulative SMM rate by 32.1%.
- Over 80% of SMM events observed within 90 days of delivery discharge occurred in the first 42 days.
- Coagulopathy (including disseminated intravascular coagulation), acute renal failure, and sepsis had the highest cumulative rates through 42 days' postdischarge.

## Introduction

Severe maternal morbidity (SMM) has been steadily increasing in recent years 1 and is associated with significant health consequences, increased risk of mortality, increased medical costs, and longer hospital stays. 2 3 Approximately two-thirds of SMM cases are considered preventable. 4 To effectively reduce SMM, improve care of SMM, and optimize system preparedness for SMM, it is essential for the United States to monitor trends over time and implement targeted interventions aimed at improving the quality of maternal care.

Existing SMM measures use health care data to identify SMM events that occur during the delivery hospitalization, with a national SMM rate of 83.9 per 10,000 for deliveries in 2019 to 2020. 5 By focusing solely on delivery hospitalizations, current SMM metrics may underestimate the true burden of maternal complications, particularly those that emerge or worsen after discharge, such as wound complications, thrombosis, sepsis, and hemorrhage. A prior study that used all-payer claims data from Massachusetts found that SMM rates increased by 22% when including prenatal and postpartum SMM-related hospitalizations. 6 Further, the existing SMM measure definition does not capture postdelivery complications treated in emergency departments (ED).

Few studies have examined postdischarge SMM using comprehensive, multistate data sources that include both inpatient and ED encounters, limiting generalizability and policy relevance. With this study, we aim to evaluate the frequency of SMM requiring hospital readmission or ED care after discharge with a federal dataset that captures all inpatient and ED visits for participating states, enabling a more complete examination of SMM events occurring after delivery discharge. We also aimed to compare different time windows for measuring postdelivery SMM and examine the types of conditions treated during each period.

## Materials and Methods

We used the 2019 to 2021 Agency for Healthcare Research and Quality (AHRQ) Healthcare Cost and Utilization Project (HCUP) State Inpatient Databases (SID) and State Emergency Department Databases (SEDD) from 17 states across four census regions. These states were the only states with valid encrypted patient identifiers available to link SID and SEDD and track individual patient utilization across settings (inpatient and ED) and years (2019–2021). We identified in-hospital deliveries among persons aged 12 to 55 years from 2019 to 2020, resulting in a study cohort of 2,551,190 deliveries. The participating states accounted for 26% of all in-hospital deliveries nationally during this period.

We estimated SMM rates at delivery using the 20-indicator AHRQ Quality Indicator Maternal Health Indicator 01 measure specifications, which exclude blood transfusions, and are fully aligned with corresponding specifications from the Centers for Disease Control and Prevention and the Alliance for Innovation on Maternal Health. 7 8 9 To capture additional SMM events that required treatment, we extended the observation period to include those events occurring up to 90 days' postdischarge (excluding transfers), identifying cases treated during a hospitalization or as a treat-and-release ED visit (visits that did not result in hospital admission). We chose a 90-day follow-up window because we wanted to focus on events proximate to the delivery. Further, this follow-up window aligns with the ICD-10-CM postpartum coding guidance (42 days) and the American College of Obstetricians and Gynecologists guidance for comprehensive postpartum visits (84 days). To ensure complete follow-up postdischarge for deliveries occurring in 2019 and 2020, we used data from 2019 to 2021.

We calculated cumulative SMM rates per 10,000 deliveries for several time points through 90 days' postdischarge. To evaluate change in rates, we calculated absolute and relative differences, compared with SMM at delivery. Further, we examined the proportion of SMM events attributable to the different indicators and timing of these indicator-specific events. All analyses were conducted in SAS 9.4 (SAS Institute, Cary, NC). Per HCUP guidance, we did not produce confidence intervals for the reported statistics because HCUP State databases contain nearly or all visits in the state, and our findings are presented specific to the study period and 17 states included in the analysis. This secondary analysis of deidentified HCUP data did not constitute research involving human participants and was exempt from institutional review board review.

## Results

Based on deliveries from 2019 to 2020 for 17 states, the SMM rate was 87.1 per 10,000 deliveries. After we included postdischarge SMM events treated in the inpatient setting in the first seven days after discharge, the rate increased by 16.8% to 101.7 per 10,000 deliveries. The SMM rate increased to 115.0 per 10,000 deliveries at 42 days after discharge, a 32.1% increase from the SMM rate calculated during the delivery hospitalization. Rates plateaued after day 42, with only a 2 to 3% increase between the subsequent time periods. Over 80% of the SMM events in the 90 days' postdischarge occurred within the first 42 days after discharge.

**Fig. 1:**
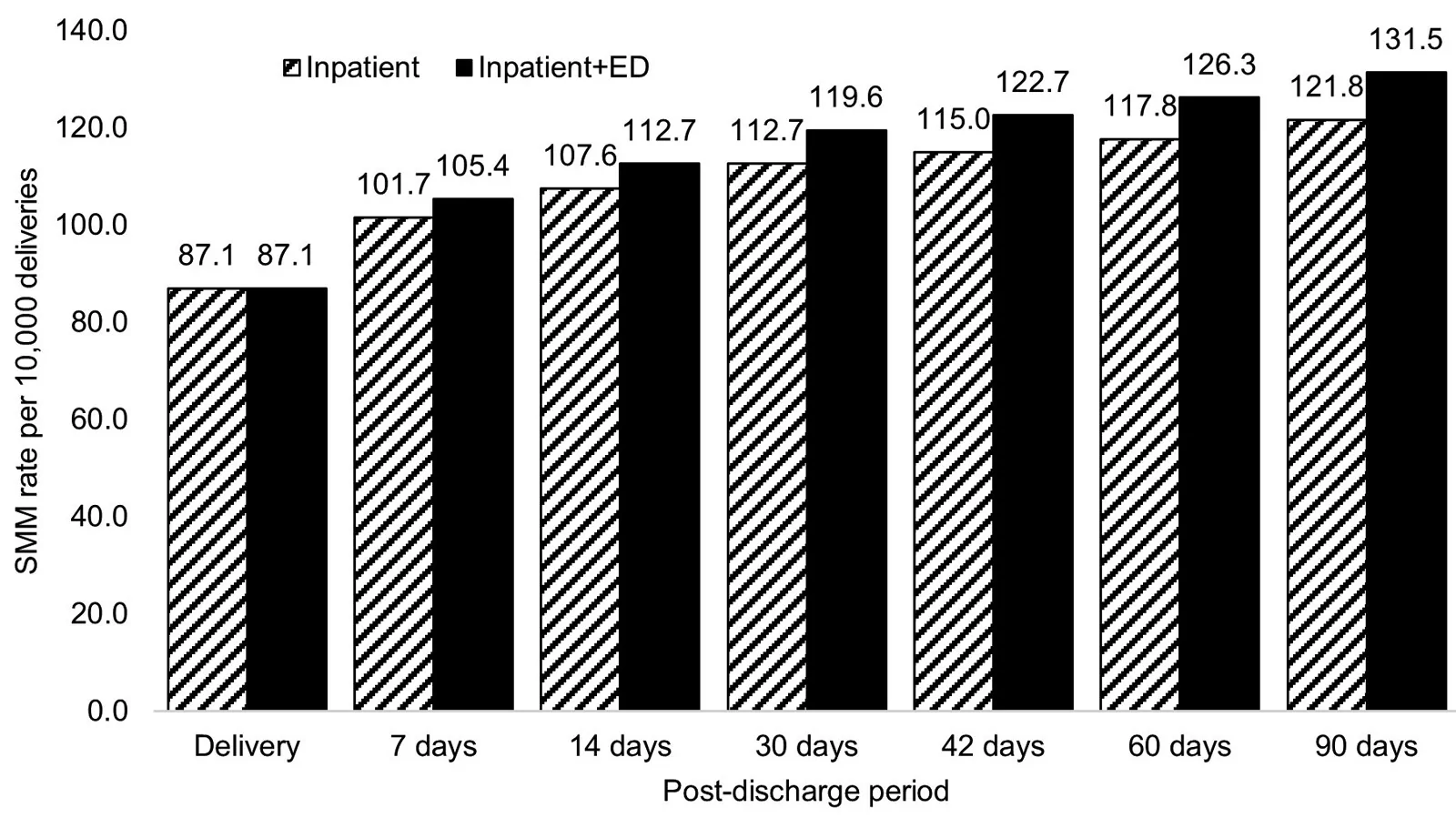
Cumulative severe maternal morbidity (SMM) rates in the postdischarge period: inpatient hospitalizations only versus inpatient hospitalizations and treat-and-release emergency department (ED) visits, 2019 to 2021. This figure displays the cumulative severe maternal morbidity or SMM rates at various time intervals (delivery, 7, 14, 30, 42, 60, and 90 days) for inpatient services alone (thatched gray bars) and for inpatient plus emergency department or ED services (dark gray bars). We observed that the cumulative SMM rate increased less than 10% with the inclusion of treat-and-release ED visits. For example, the SMM rate at 42 days' postdischarge was 122.7 per 10,000 deliveries, 6.7% higher than the inpatient-only rate. The analysis includes data from 17 states with linkable inpatient and ED records. Source: Agency for Healthcare Research and Quality (AHRQ) Healthcare Cost and Utilization Project (HCUP), State Inpatient Databases, State Emergency Department Databases, 17 States, 2019 to 2021.

When we included treat-and-release ED visits, the SMM rate at 42 days' postdischarge was 122.7 per 10,000 deliveries, 6.6% higher than the inpatient-only rate ( Fig. 1 ). We found that 78.6% of postdischarge SMM events in the first 42 days after discharge were treated in the inpatient setting.

**Fig. 2:**
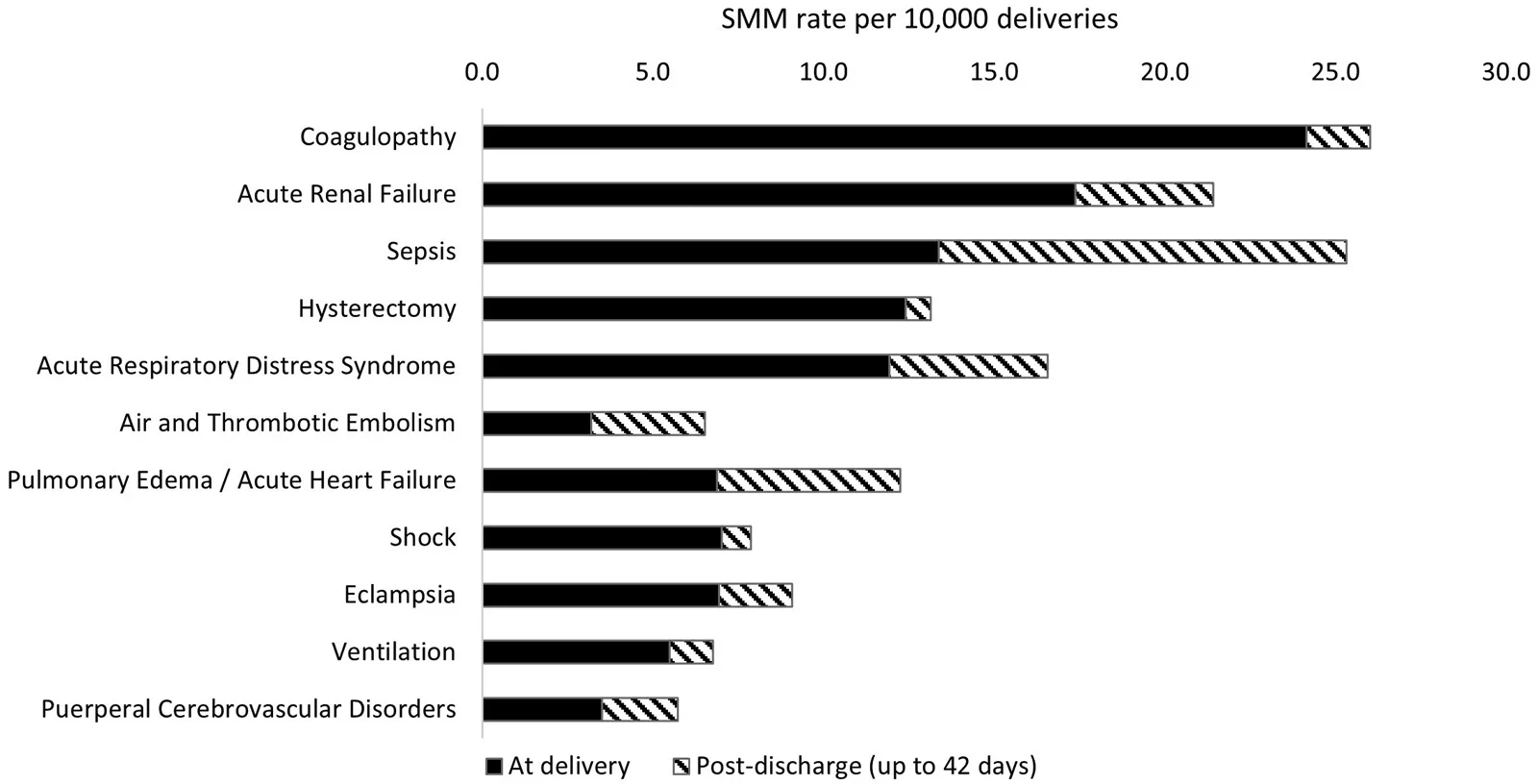
Cumulative severe maternal morbidity (SMM) rates in the postdischarge period by indicator and time period, 2019 to 2021. This figure presents the rates (per 10,000 deliveries) of selected SMM indicators occurring at delivery (dark gray bars) versus during the postdischarge period at up to 42 days' postpartum (thatched gray bars). Coagulopathy (including disseminated intravascular coagulation), acute renal failure, and sepsis had the highest cumulative rates through 42 days' postdischarge, compared with other indicators. Coagulopathy, hysterectomy, and shock predominantly occurred at delivery, whereas sepsis and air or thrombotic embolism occurred at both time points (delivery and postdischarge). The analysis includes data from 17 states with linkable inpatient and emergency department records. Source: Agency for Healthcare Research and Quality (AHRQ) Healthcare Cost and Utilization Project (HCUP), State Inpatient Databases, State Emergency Department Databases, 17 States, 2019 to 2021.

We looked at rates for the specific indicators that comprised the SMM measure and found that coagulopathy (including disseminated intravascular coagulation; 26.0 per 10,000 deliveries), acute renal failure (21.4 per 10,000 deliveries), and sepsis (25.3 per 10,000 deliveries) had the highest cumulative rates through 42 days' postdischarge, compared with other indicators ( Fig. 2 ). Coagulopathy, hysterectomy, and shock predominantly occurred at delivery, whereas sepsis and air or thrombotic embolism occurred equally at both time points (delivery and postdischarge).

To identify the most common indicators of SMM at different time points, we compared the proportion of SMM events attributed to each indicator at delivery versus after discharge ( Table 1 ). Among individuals with SMM indicated at delivery, the most frequent indicators were coagulopathy (27.7%), acute renal failure (20.0%), and sepsis (15.4%). In contrast, among those with SMM identified postdischarge, sepsis (34.4%), pulmonary edema/acute heart failure (15.4%), and acute respiratory distress syndrome (13.4%) were most common in the 42 days after discharge.

**Table 1:** Proportion of severe maternal morbidity events attributable to each indicator: at delivery versus in the first 42 days' postdischarge period, 2019 to 2021

SMM indicator	Among deliveries with any SMM at delivery ( *n* = 22,211)	Among deliveries with any SMM in first 42 days' postdischarge ( *n* = 7,140)
Coagulopathy	27.7	5.4
Acute renal failure	20.0	11.7
Sepsis	15.4	34.4
Hysterectomy	14.2	2.1
Acute respiratory distress syndrome	13.7	13.4
Shock	8.1	2.4
Eclampsia	8.0	6.2
Pulmonary edema/acute heart failure	7.9	15.4
Ventilation	6.3	3.7
Puerperal cerebrovascular disorders	4.0	6.4
Air and thrombotic embolism	3.7	9.6

Abbreviation: SMM, severe maternal morbidity.

Note. This table only includes indicators with a proportion of greater than 1% at delivery and in the 42 postdischarge period (11 out of 20 SMM indicators).

Source: Agency for Healthcare Research and Quality (AHRQ) Healthcare Cost and Utilization Project (HCUP) State Inpatient Databases, State Emergency Department Databases, 17 States, 2019 to 2021.

## Discussion

We found the risk of rehospitalization for SMM was highest in the first 7 days after discharge, and over 80% of postdischarge SMM cases within 90 days were captured in the first 42 days. Previous studies reported smaller increases in the SMM rate using a 42-day window; however, these studies were from earlier time periods, limited in geographic scope, or based on claims data that excluded the uninsured population. 6 These other studies also focused exclusively on inpatient hospitalizations after discharge. In our study, we found that including SMM events treated in the ED setting resulted in an incremental increase compared with SMM rates based only on inpatient records. The discrepancy between ED visits and inpatient readmissions with SMM may reflect patients who could not be linked in the data due to differing identifiers or transfers to out-of-state or federal hospitals excluded from the hospital discharge data provided by participating states.

Finally, the at-delivery rates for the individual indicators that comprise the SMM measure were similar to rates reported elsewhere from the same time period. 1 The SMM indicators most common in the postdischarge period reflect subacute complications from birth and the postpartum period, such as infection, venous thromboembolism, and pulmonary edema. Recognizing the burden of SMM in the early postpartum period after discharge from the delivery hospitalization may also inform improvements to the postpartum care model in those critical weeks following discharge, enabling earlier recognition and management of complications before they progress to SMM.

There are several potential limitations of this study. While the full HCUP SID includes 48 states and the District of Columbia, our analysis was limited to 17 states with valid encrypted patient identifiers to support linkage, and these states represent around one-quarter of all inpatient deliveries in the United States. The SMM rates from this study were similar to the national rates produced by HCUP, 5 supporting the generalizability of our findings. Our SMM rates were not risk-adjusted; however, the most common risk adjustment variables—such as age and sex—remain stable over the 90-day postpartum period and are unlikely to affect our primary outcome, which focused on changes in SMM rates over time. Because patient identifiers are state-specific, postdischarge SMM events that occurred in a different state from the delivery location could not be linked in the HCUP data, potentially underestimating postdischarge rates. However, the impact of this limitation should be minimal; the cumulative SMM rate through 42 days' postdischarge changed by only 0.5% when we excluded women who delivered outside their state of residence. Finally, while the definition of SMM used in this study is extensively validated and used, there are limitations to SMM measures defined using diagnosis and procedure codes. 10

Our analysis included data from 2020—a year significantly impacted by the coronavirus disease 2019 (COVID-19) public health emergency, which disrupted health care utilization patterns. It is possible that SMM rates following discharge from the delivery hospitalization were altered during this period due to pandemic-related factors such as delayed care-seeking, greater use of telehealth, and health care system strain. The long-term effects of the COVID-19 pandemic on postpartum health care utilization remain to be fully understood.

Our study highlights the importance of capturing SMM in the postpartum period, particularly within the first 42 days after delivery discharge. In 2018, the American College of Obstetricians and Gynecologists placed a renewed focus on the fourth trimester and proposed a new paradigm for postpartum care, recommending ongoing contact between patients and their providers starting within 3 weeks of delivery. 11 Our findings showing a significant occurrence of SMM in the first 6 weeks' postpartum support this recommendation. Accurately measuring the occurrence of SMM also aligns with national, state, and local efforts supported by the Alliance for Innovation on Maternal Health and is an important step toward better understanding the scale of the problem, promoting safe obstetric care and safe discharge practices, and evaluating the effectiveness of postpartum care interventions. Perinatal quality efforts should focus on severe outcomes associated with labor and delivery, both during the delivery hospitalization and in the 6 weeks after discharge.
